# In Silico and In Vitro Identification of Pan-Coronaviral Main Protease Inhibitors from a Large Natural Product Library

**DOI:** 10.3390/ph15030308

**Published:** 2022-03-03

**Authors:** Nasim Shahhamzehei, Sara Abdelfatah, Thomas Efferth

**Affiliations:** Department of Pharmaceutical Biology, Institute of Pharmaceutical and Biomedical Sciences, Johannes Gutenberg University, Staudinger Weg5, 55128 Mainz, Germany; nshahham@uni-mainz.de (N.S.); saabdelf@uni-mainz.de (S.A.)

**Keywords:** infectious diseases, natural products, pan-inhibitor, virtual drug screening

## Abstract

The main protease (M^pro^ or 3CL^pro^) in coronaviruses represents a promising specific drug target as it is essential for the cleavage of the virus polypeptide and has a unique cleavage site that does not exist in human host proteases. In this study, we explored potential natural pan-coronavirus drugs using in vitro and in silico approaches and three coronavirus main proteases as treatment targets. The PyRx program was used to screen 39,442 natural-product-like compounds from the ZINC database and 121 preselected phytochemicals from medicinal plants with known antiviral activity. After assessment with Lipinski’s rule of five, molecular docking was performed for the top 33 compounds of both libraries. Enzymatic assays were applied for the top candidates from both in silico approaches to test their ability to inhibit SARS-CoV-2 M^pro^. The four compounds (hypericin, rosmarinic acid, isorhamnetin, and luteolin) that most efficiently inhibited SARS-CoV-2 M^pro^ in vitro were further tested for their efficacy in inhibiting M^pro^ of SARS-CoV-1 and MERS-CoV. Microscale thermophoresis was performed to determine dissociation constant (Kd) values to validate the binding of these active compounds to recombinant M^pro^ proteins of SARS-CoV-2, SARS-CoV-1, and MERS-CoV. The cytotoxicity of hypericin, rosmarinic acid, isorhamnetin, and luteolin was assessed in human diploid MRC-5 lung fibroblasts using the resazurin cell viability assay to determine their therapeutic indices. Sequence alignment of M^pro^ of SARS-CoV-2 demonstrated 96.08%, 50.83%, 49.17%, 48.51%, 44.04%, and 41.06% similarity to M^pro^ of other human-pathogenic coronaviruses (SARS-CoV-1, MERS-CoV, HCoV-NL63, HCoV-OC43, HCoV-HKU1, and HCoV-229E, respectively). Molecular docking showed that 12 out of 121 compounds were bound to SARS-CoV-2 M^pro^ at the same binding site as the control inhibitor, GC376. Enzyme inhibition assays revealed that hypericin, rosmarinic acid, isorhamnetin, and luteolin inhibited M^pro^ of SARS-CoV-2, while hypericin and isorhamnetin inhibited M^pro^ of SARS-CoV-1; hypericin showed inhibitory effects toward M^pro^ of MERS-CoV. Microscale thermophoresis confirmed the binding of these compounds to M^pro^ with high affinity. Resazurin assays showed that rosmarinic acid and luteolin were not cytotoxic toward MRC-5 cells, whereas hypericin and isorhamnetin were slightly cytotoxic. We demonstrated that hypericin represents a potential novel pan-anti-coronaviral agent by binding to and inhibiting M^pro^ of several human-pathogenic coronaviruses. Moreover, isorhamnetin showed inhibitory effects toward SARS-CoV-2 and SARS-CoV-1 M^pro^, indicating that this compound may have some pan-coronaviral potential. Luteolin had inhibitory effects against SARS-CoV-2 M^pro^.

## 1. Introduction

Coronaviruses are enveloped, positive-sense, single-stranded RNA viruses with different hosts occurring in avians and mammals. This family is divided into four genera: α-, β-, γ-, and δ-coronaviruses. Among them, seven coronaviruses are pathogenic to humans, i.e., HCoV-229E and HCoV-NL63, which belong to the α-coronaviruses, and HCoV-OC43, HKU1, SARS-CoV-1, MERS-CoV, and the novel coronavirus nominated as SARS-CoV-2, which belong to the β-coronaviruses [1,2]. SARS-CoV-1, MERS-CoV, and SARS-CoV-2 are highly pathogenic and cause viral pneumonia in patients. Meanwhile, the other four coronaviruses (HCoV-229E, HCoV-NL63, HCoV-OC43, and HKU1) usually infect the upper human respiratory system and cause the common cold (5–30%) [3]. However, they can also cause severe or lethal diseases in patients who are children, elderly, or immunodeficient [4].

The period between disease outbreaks has become shorter, and there is a possibility that more viral epidemics will occur soon. As vaccines provoke immunological memory against specific antigens, it is probable that the current therapeutic strategies targeting SARS-CoV-2 and its mutant variants may be inefficient against future coronaviruses that emerge in the human population. This threat is supported by data showing that vaccines formulated against SARS-CoV-1 antigens do not effectively protect from infections related to other SARS-like coronaviruses that are currently circulating in bat populations [5]. However, novel bat coronaviruses have sequence homologies of more than 90% to SARS-CoV-2 [6,7]. Hence, it is likely that more still-undetected bat coronaviruses with high similarity to SARS-CoV-2 exist and that some of them may have the potential for starting the outbreak of the next coronavirus epidemic or pandemic. It is common sense among the virological community that COVID-19 may not be the last pandemic and that more will threaten us in the future [8,9]. Consequently, there is an urgent need to identify and develop pan-anti-coronaviral drugs to be better prepared for the next coronavirus pandemic compared with our preparation for the current one. 

Sequence comparison studies showed that SARS-CoV-2 shares approximately 79% sequence similarity with SARS-CoV-1 and approximately 50% with MERS-CoV [10]. Moreover, SARS-CoV-2 has a similar genome organization compared to β-coronaviruses with 14 open reading frames (ORFs). The large reading frame, ORF-1ab, encodes two polyproteins, pp1a and pp1ab, that are cleaved into 16 nonstructural proteins (nsp1-16, also termed replicase complex) by the main protease (at 11 positions), implying its important role in viral replication. This main protease is located in nsp5 and the papain-like protease (PL^pro^) in nsp3. The rest of ORF-1ab encodes nine accessory proteins and four structural proteins, i.e., spike (S), envelope (E), membrane (M), and nucleocapsid (N) [11,12]. The coronavirus main protease (M^pro^, also termed 3CL^pro^) is a cysteine protease with cysteine^145^ and histidine^41^ in its active site [10]. Its structure is composed of two monomers, and each of them consists of three domains. Domain I (residues 8–101) and domain II (residues 102–184) are catalytic domains and have an antiparallel β-barrel. Domain III (residues 201–303) is responsible for enzyme dimerization and has five α-helices [13,14]. M^pro^ has a unique cleavage site at conserved Leu-Gln↓ (Ser/Ala/Gly) [15,16]. This feature is absent in closely related human host proteases, and the side effects of M^pro^ inhibitors in human patients are limited. Therefore, the main protease represents an ideal target for developing anti-coronaviral drugs [17].

Natural products provide a rich resource for novel antiviral compounds. They are an extensive source of oral drugs based on Lipinski’s rule of five [18]. Furthermore, they are evolutionarily optimized for interaction with different proteins and biological targets, which explains their high relevance for a variety of therapeutic purposes. Natural products have been used in traditional medicine for centuries, which provides insights regarding efficacy and safety. While many natural products have been extracted, only a few have been marketed as drugs. Therefore, further identification of their active compounds is useful for the treatment of viral infections and the management of outbreaks [19,20]. 

In this investigation, we aimed to explore potential pan-coronavirus compounds using in vitro and in silico approaches against several coronavirus M^pro^. We studied 39,442 natural-product-like compounds from the ZINC database and 121 preselected natural products from medicinal plants with known antiviral activity [21,22,23,24,25,26,27] by virtual drug screening using SARS-CoV-2 M^pro^ as a target to identify lead compounds that may be further developed as pan-coronaviral drugs. Then, the top 12 compounds identified in silico were investigated for the inhibition of SARS-CoV-2 M^pro^ in vitro. The top four compounds from this experiment were used to calculate IC_50_ values for the orthologous main proteases of SARS-CoV-1 and MERS-CoV. Finally, microscale thermophoresis was used as a biochemical assay to verify the binding of these compounds to recombinant M^pro^.

## 2. Results

### 2.1. In Silico Studies 

**Figure 1:**
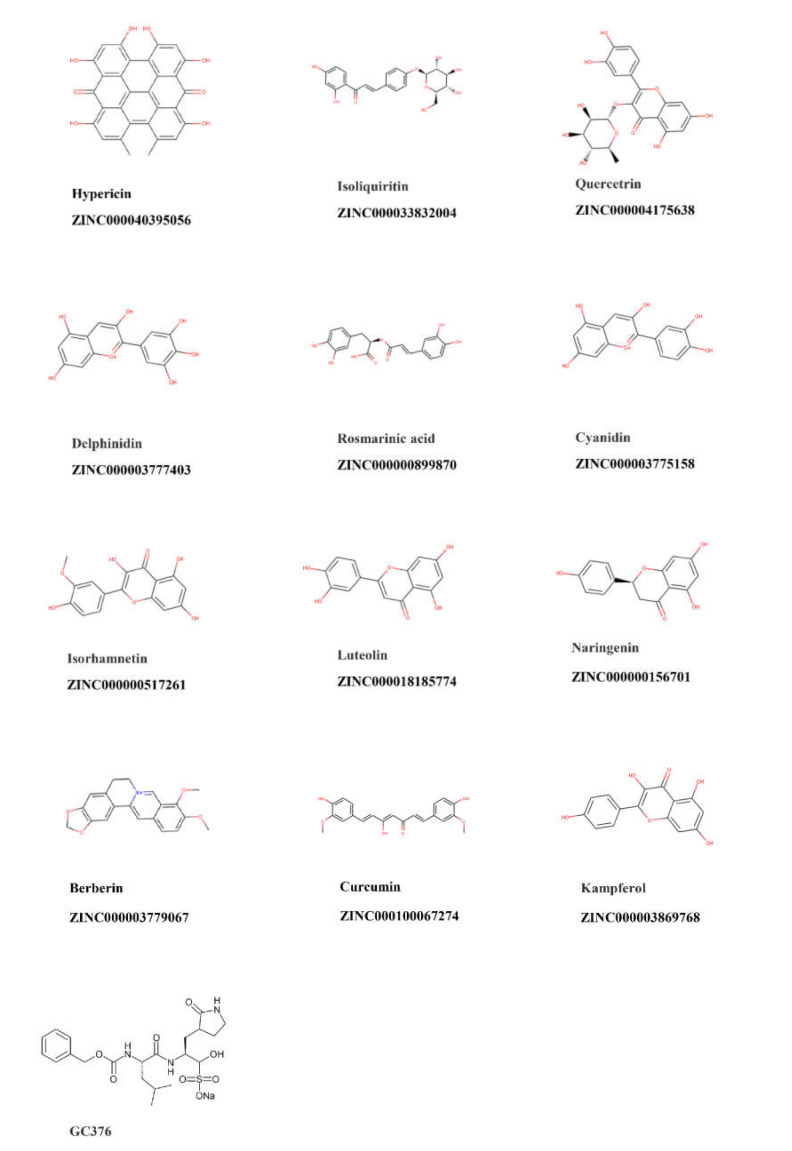
Chemical structures of the top 12 compounds with the lowest binding energy to SARS-CoV-2 main protease.

**Figure 2:**
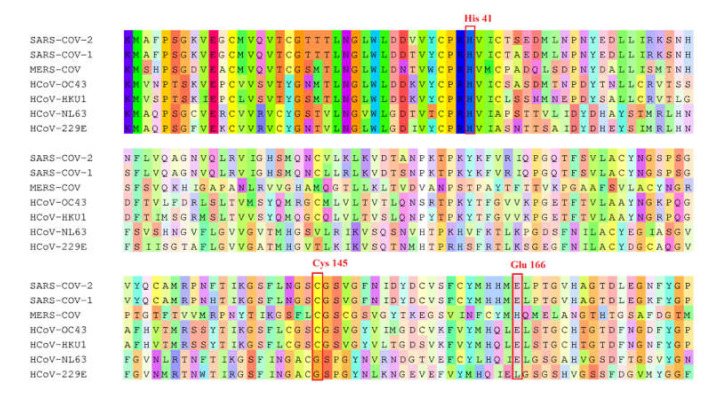
Sequence alignments of the main protease (binding site sequence) among SARS-CoV-2, SARS-CoV-1, MERS-CoV, HcoV-OC43, HcoV-HKU1, HcoV-NL63, and HcoV-299E. Catalytic residues are indicated by the red box.

By using a ZINC library of 39,442 natural-product-like compounds and a second natural product library of 121 compounds that were preselected from medicinal plants with known antiviral activity, we performed virtual screening with PyRx. A total of 89 hits from the ZINC natural-product-like library and 32 hits from the antiviral natural product library were selected on the basis of their lowest PyRx-based binding energies to SARS-CoV-2 M^pro^. These compounds were assessed using the Lipinski rules, and compounds with a molecular weight ≤ 500 and an octanol–water partition coefficient of log *p* ≤ 5 were considered for further investigation, i.e., 21 candidate compounds from the ZINC database and 12 candidates from the antiviral natural product library. These 33 compounds were subjected to molecular docking with AutoDock 4.2.6. The lowest binding energy values (LBEs) and predicted inhibition constants (pKi’s) of the 12 candidates from the antiviral natural product library that were used for subsequent experiments are shown in Table 1. Figure 1 depicts the structures of these 12 compounds and the known M^pro^ inhibitor, GC376, which was used as a positive control. To identify similarities between the main protease of seven human coronaviruses, we performed protein alignments. The results revealed 96.08%, 50.83%, 49.17%, 48.51%, 44.04%, and 41.06% identity of SARS-CoV-2 M^pro^ to SARS-CoV-1, MERS-CoV, HCoV-NL63, HcoV-OC43, HcoV-HKU1, and HcoV-229E, respectively (Table 2). Figure 2 shows the highly conserved amino acid residues between the seven human coronaviruses.

### 2.2. Inhibition of M^pro^ Enzyme Activity 

**Figure 3:**
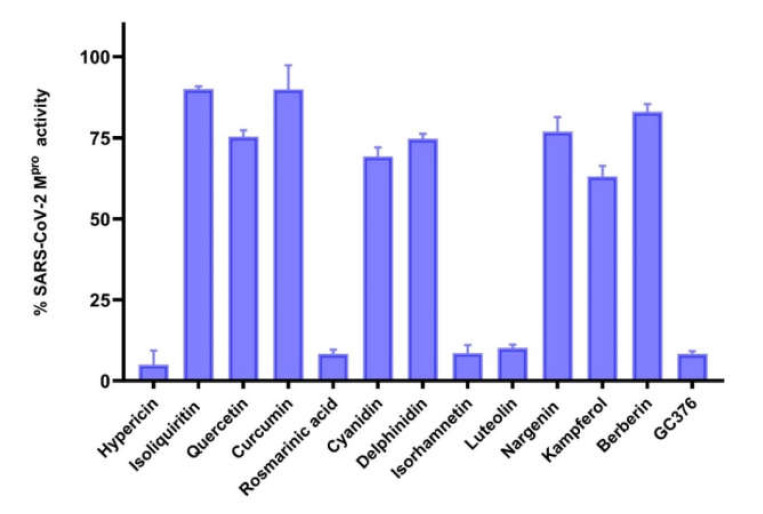
Percent activity of SARS-CoV-2 main protease in the presence of 12 compounds (100 µM). GC376 was used as a positive control. The results are expressed as mean value ± standard deviation.

**Figure 4:**
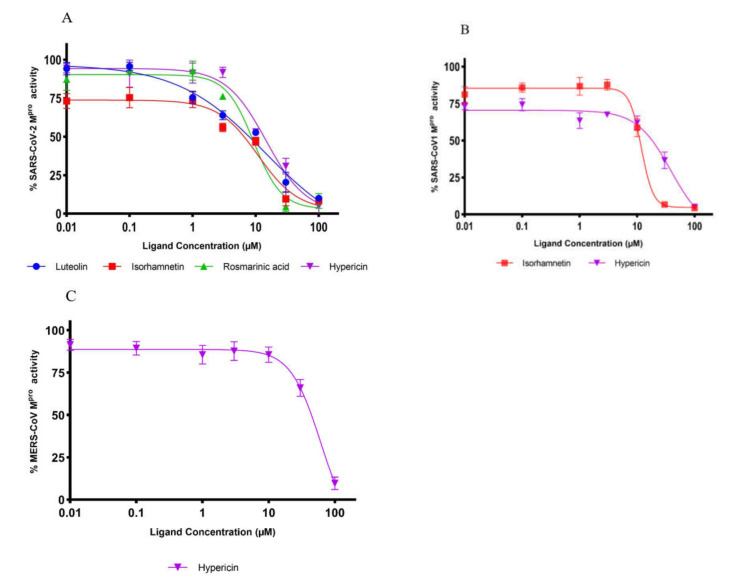
Dose-dependent inhibition of (**A**) SARS-CoV-2, (**B**) SARS-CoV-1, and (**C**) MERS-CoV main protease activity. Triplicate experiments were performed for each compound, and the IC_50_ values are presented as mean ± standard deviation (SD).

We performed in vitro enzymatic assays to validate whether the compounds selected from the in silico studies inhibit the activity of M^pro^ of SARS-CoV-2. As expected, hypericin, rosmarinic acid, isorhamnetin, and luteolin were the most active natural products among the 12 compounds preselected by our bioinformatical approach. These compounds inhibited enzymatic activity by more than 50% (Figure 3). Therefore, these four compounds were subjected to subsequent dose-response experiments to calculate the concentration of each compound required to inhibit M^pro^ activity by half (IC_50_). The percentage of activity versus the log concentration of the inhibitors was used to calculate the IC_50_ values (Figure 4A–C). The IC_50_ values for hypericin, rosmarinic acid, isorhamnetin, and luteolin for SARS-CoV-2 CL^pro^ were 23.30, 9.43, 8.42, and 11.81 µM, respectively. The IC_50_ values for the inhibition of M^pro^ of SARS-CoV-1 by hypericin and isorhamnetin were 19.43 and 13.13 µM, respectively. Rosmarinic acid and luteolin inhibited the enzymatic activity of M^pro^ of SARS-CoV-1 only at the highest concentration of 100 µM by 31% and 44%, respectively. The IC_50_ value for the inhibition of MERS-CoV M^pro^ by hypericin was 49.65 µM. At a concentration of 100 µM, rosmarinic acid, luteolin, and isorhamnetin inhibited MERS-CoV M^pro^ activity to 14.9%, 21.3%, and 26.3%, respectively (Table 3). 

### 2.3. Microscale Thermophoresis

**Figure 5:**
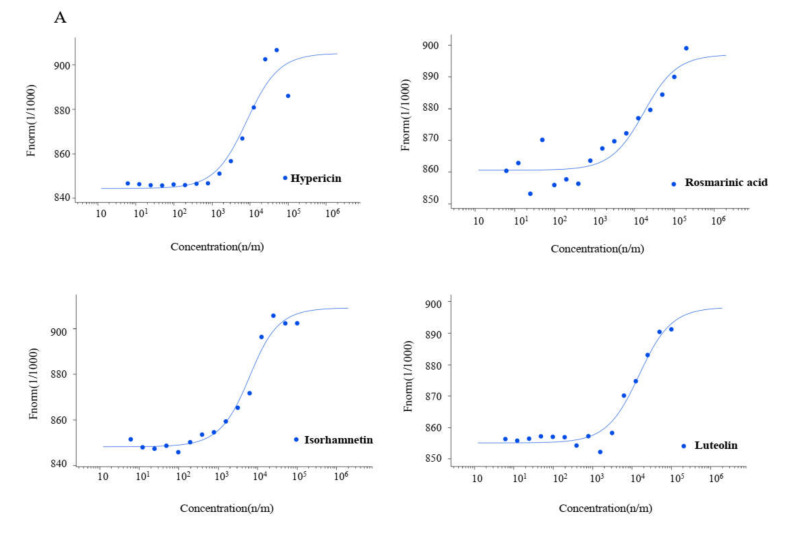

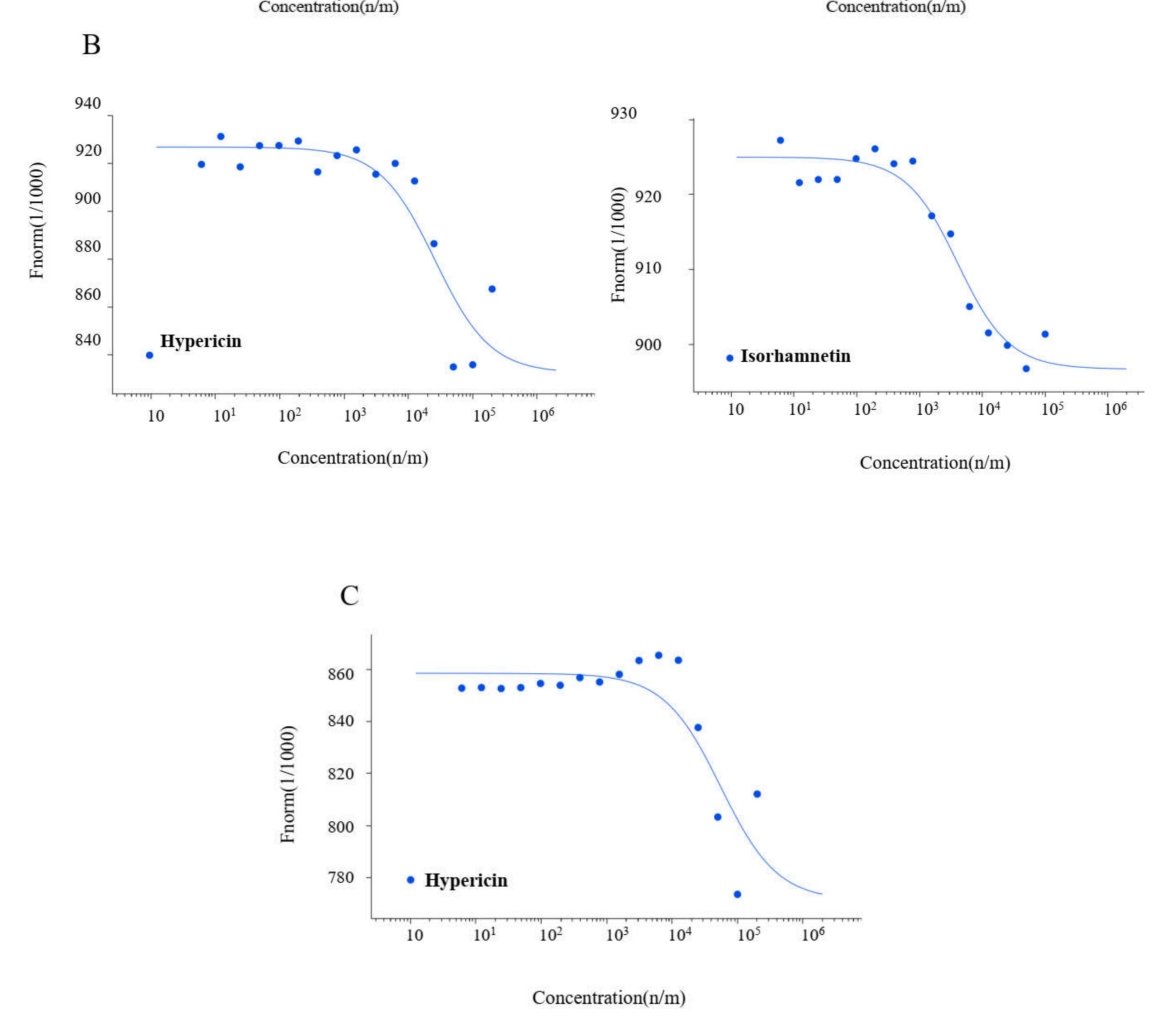
Binding of inhibitors to (**A**) SARS-CoV-2, (**B**) SARS-CoV-1, and (**C**) MERS-CoV main protease as measured by MST.

Microscale thermophoresis is a sensitive technique used to determine the binding between unlabeled molecules and labeled macromolecules (i.e., proteins). The labeled recombinant M^pro^ of SARS-CoV-2, SARS-CoV-1, and MERS-CoV were titrated against different concentrations of the selected compounds (Figure 5A–C). Hypericin, rosmarinic acid, isorhamnetin, and luteolin were bound in vitro to SARS-CoV-2 M^pro^ with Kd values of 7.73, 15.47, 4.379, and 13.417 µM, respectively. This was also the case with M^pro^ of the other coronavirus family members. SARS-CoV-1 M^pro^ was inhibited with Kd values of 25.49 µM by hypericin and 3.60 µM by isorhamnetin. MERS-CoV M^pro^ was inhibited with a Kd value of 54.91 µM by hypericin (Table 4).

### 2.4. Binding of the Top Candidates

**Figure 6:**
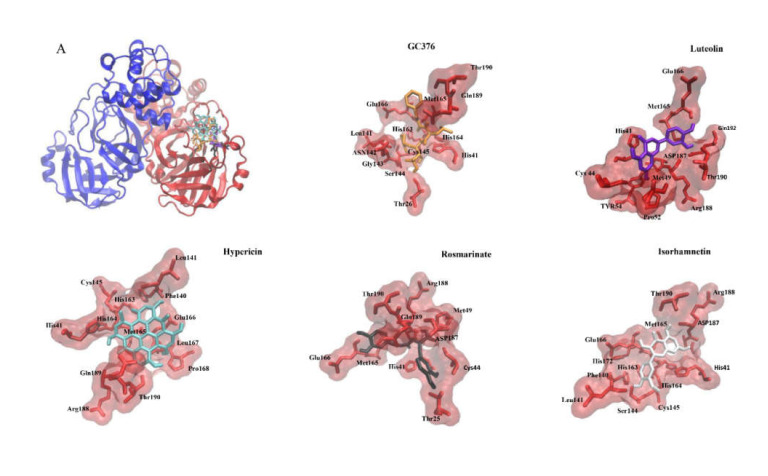

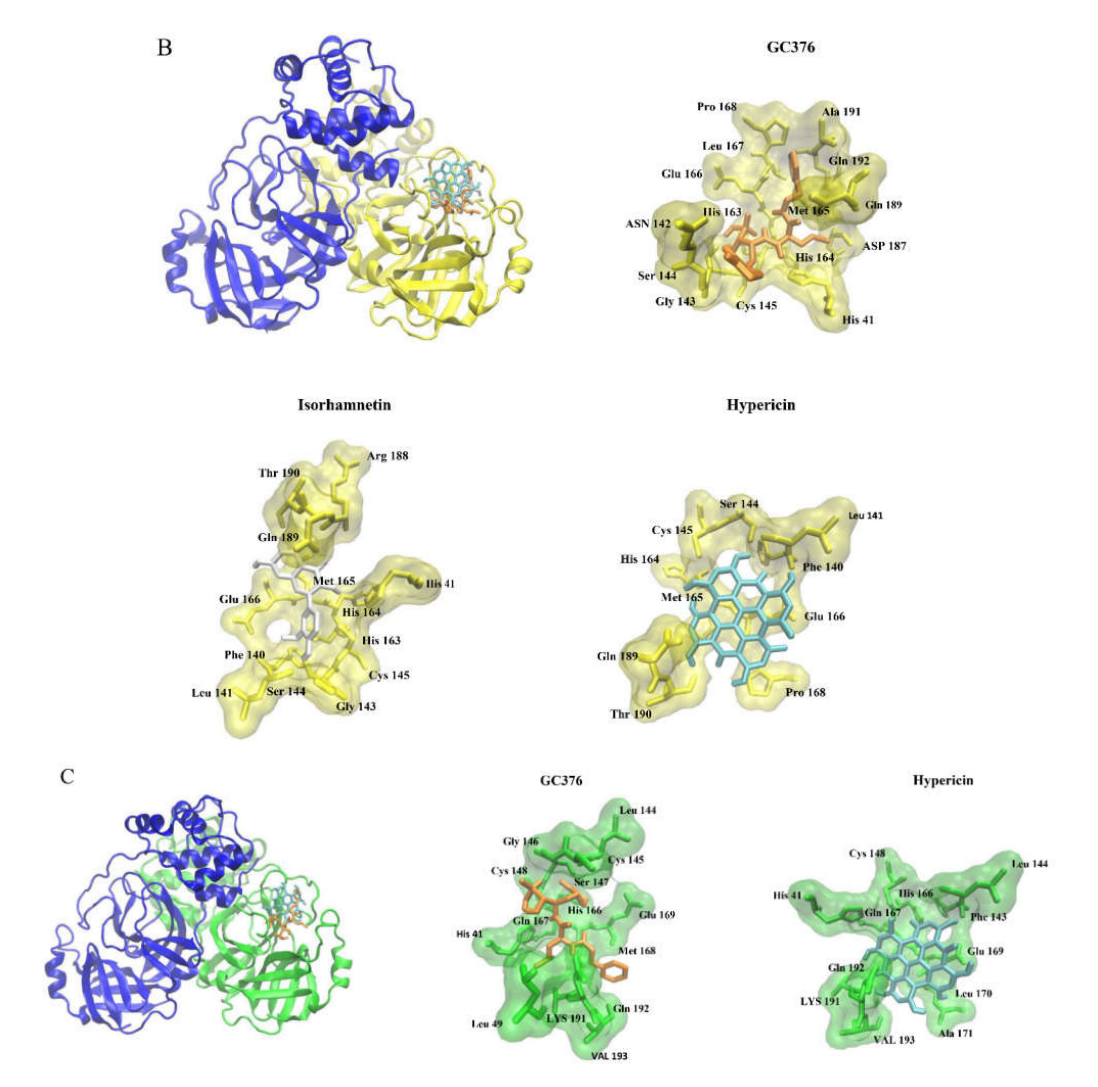
Molecular docking of potential inhibitors and GC376 (positive control) to the binding site of (**A**) SARS-CoV-2 M^pro^ (PDB ID: 6XMk), (**B**) SARS-CoV-1 M^pro^ (PDB ID:6xhl), and (**C**) MERS-CoV M^pro^ (PDB ID:6vh0).

**Figure 7:**
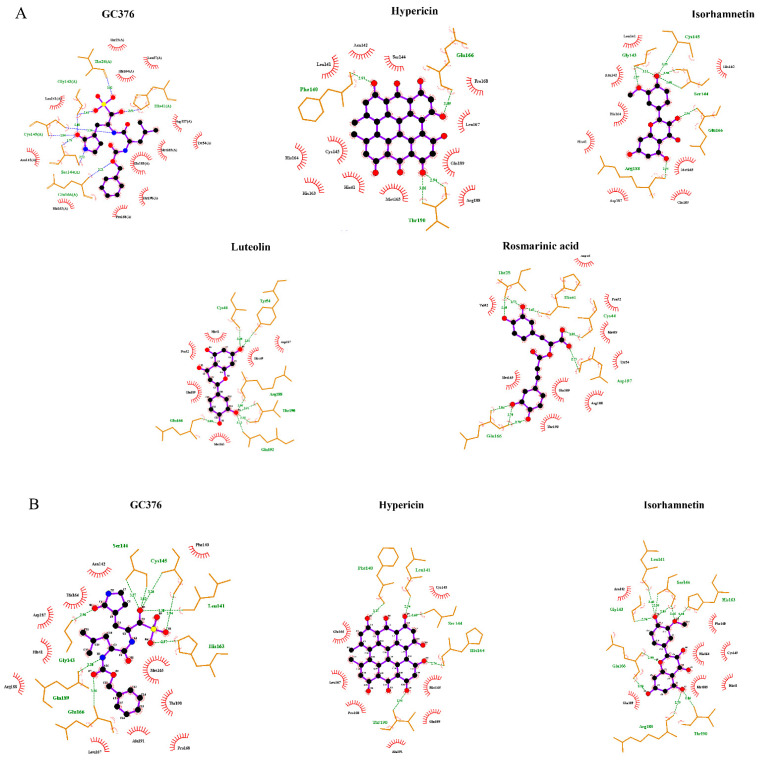

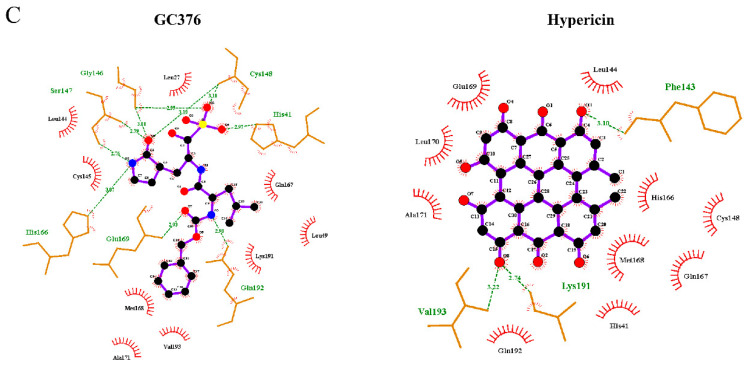
Two-dimensional representations of inhibitor interactions with (**A**) SARS-CoV-2 M^pro^, (**B**) SARS-CoV-1 M^pro^, and (**C**) MERS-CoV M^pro^ were analyzed using Ligplot. Hydrogen bonds are shown as green dotted lines, while the spoked arcs represent residues forming hydrophobic interactions with the ligands.

Molecular docking in silico revealed high binding affinities of the best candidate compounds and the control inhibitor, GC376, to M^pro^ of seven human coronaviruses (Table 5). Figure 6A–C shows the molecular interactions of potential inhibitors and GC376 with SARS-CoV-2, SARS-CoV-1, and MERS-CoV M^pro^. The best candidate compounds shared the same binding site at M^pro^ as the control inhibitor, GC376. Hypericin, rosmarinic acid, isorhamnetin, luteolin, and the control inhibitor, GC376, formed hydrogen bonds or hydrophobic interactions with at least one of the catalytic residues (Cys^145^, His^41^) in the binding site of M^pro^ of SARS-CoV-2, SARS-CoV-1, and MERS-CoV (Figure 7A–C). Moreover, these compounds interacted with Glu1^166^ of SARS-CoV-2 and SARS-CoV-1 and Glu^169^ of MERS-CoV, all of which play an important role in the dimerization of M^pro^ [28,29,30,31].

### 2.5. Cell Viability Assay

**Figure 8:**
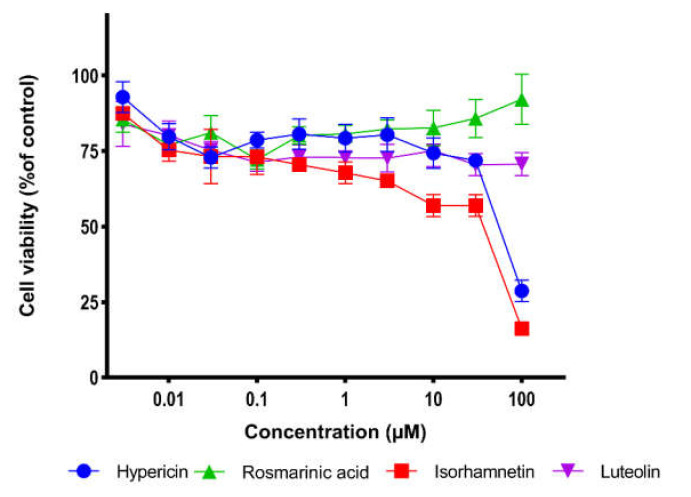
Dose-response curves of candidate compounds against MRC-5 cell line. Data are shown as mean values ± standard deviations of three independent experiments by the resazurin assay.

The inhibitory effects on the cell viability of the potential M^pro^ inhibitors toward human MRC-5 fibroblasts were assessed using the resazurin assay. As shown in Figure 8, luteolin and rosmarinic acid did not show significant inhibitory effects within the tested concentration range. Hypericin and isorhamnetin showed a slight inhibition of viability of MRC-5 cells with CC_50_ values of 55.46 ± 2.2 µM and 36.80 ± 3.4 µM, respectively (Table 6). The therapeutic indices of hypericin for SARS-CoV-2, SARS-CoV-1, and MERS-CoV M^pro^ were 2.38, 2.85, and 1.11, respectively. For isorhamnetin, the therapeutic index was 4.37 and 2.8 for SARS-CoV-2 and SARS-CoV-1, respectively (Table 7).

## 3. Discussion

During the past two decades, highly infectious pathogens rapidly emerged, such as SARS-CoV-1 in 2003, MERS-CoV in 2012, and SARS-CoV-2 at the end of 2019. Therefore, there is an urgent need to investigate new broad-spectrum anti-CoVs drugs. The main protease was proposed as a promising target for the development of pan-coronaviral drugs as it significantly differs from human proteases and is highly conserved between coronavirus family members [16]. 

In this study, we first performed a computer-based approach to screen 39,442 natural-product-like compounds from the ZINC database and 121 preselected natural products from medicinal plants with known antiviral activity to find candidate compounds with a high binding affinity to SARS-CoV-2 M^pro^. As a result of PyRx-based virtual drug screening, assessment using the Lipinski rule of five, and molecular docking using AutoDock 4.2.6, 33 compounds were selected and subjected to AutoDock to validate the virtual screening results. Twelve compounds were selected for further in vitro experiments. To analyze whether these 12 compounds affect SARS-CoV-2 M^pro^ activity in vitro, we performed M^pro^ enzyme activity inhibition assays. Hypericin, rosmarinic acid, isorhamnetin, and luteolin inhibited SARS-CoV-2 M^pro^. The binding site of SARS-CoV-2 includes a catalytic dyad (His41 and Cys145) and several subsites (S1–S5). The S1 subunit consists of His163, Glu166, Cys145, Gly143, His172, and Phe140. The S2 subunit comprises Cys145, His41, and Thr25. S3–S5 consists of Met165, Met49, His41, Glu166, and Gln189. These subunits play a key role in substrate binding [32,33]. Molecular docking revealed that hypericin, rosmarinic acid, isorhamnetin, and luteolin not only bound to M^pro^ through these subunits but also interacted (hydrophobic or hydrogen binding) with at least one of the catalytic center residues (His41 and Cys145) (Figure 7A). Moreover, microscale thermophoresis confirmed the binding of these four natural compounds to SARS-CoV-2 M^pro^. Although the K_d_ values were different, all compounds showed a high binding affinity to SARS-CoV-2 M^pro^. Consequently, we concluded that the in silico data reflected the in vitro situation as there was a good correlation between the computationally predicted lowest binding energies (−12.44, −9.98, −9.06, and −9.01 kcal/mol) and the experimentally measured percentages of activity (4.95%, 8.28%, 8.56%, and 10.10%) of SARS-CoV-2 M^pro^ in the presence of hypericin, rosmarinic acid, isorhamnetin, and luteolin, respectively. To develop a potential pan-HCoV inhibitor, we also performed M^pro^ enzyme activity inhibition assays for SARS-CoV-1 and MERS-CoV. Hypericin and isorhamnetin inhibited SARS-CoV-1 M^pro^, while only hypericin inhibited MERS-CoV M^pro^. Microscale thermophoresis confirmed that these two compounds were bound to SARS-CoV-1 and MERS-CoV M^pro^ with high affinities.

Cell viability assays showed that luteolin and rosmarinic acid did not inhibit human fetal MRC-5 lung fibroblasts in the highest concentration tested (100 µM) while hypericin and isorhamnetin showed slight toxicity. 

Hypericin is a natural polyquinone from *Hypericum perforatum* (St. John’s wort) and is traditionally used as an anti-depressive and wound-healing drug [34]. Hypericin has antitumor, antivirus, and anti-depression activity. It exhibits in vitro activity against infectious bronchitis virus (IBV) by the inhibition of apoptosis in host cells and the production of reactive oxygen species [35]. Additionally, hypericin inhibits hepatitis C virus (HCV) replication via downregulation of heme oxygenase-1 expression and deacetylation in vitro [36]. However, hypericin caused phytotoxicity without detectable anti-HCV activity in patients with chronic HCV infection who were provided oral doses of 0.05 and 0.10 mg/kg/d [37]. Although hypericin inhibits human immunodeficiency virus (HIV) in vitro and in vivo [38,39], a clinical trial revealed phytotoxicity of orally administered hypericin (0.5 mg/kg daily) without antiretroviral activity in a limited number of patients [40]. Moreover, hypericin inhibits the replication of α-coronaviruses (PEDV and TGEV) through the inhibition of M^pro^ [41]. Hypericin inhibits M^pro^ of SARS-CoV-2 with a CC_50_ value of 63.6 µM [42]. Our in silico and in vitro results indicated that hypericin both binds and inhibits M^pro^ of β-coronaviruses. Previous in silico and in vitro studies showed that hypericin has anti-inflammatory activity [43,44,45] and is a potential treatment for rheumatoid arthritis [44]. Thus, hypericin is a promising pan-CoV inhibitor that, due to its anti-inflammatory effects, may be used in coronavirus-infected patients suffering from autoimmune reactions (“long COVID”) who are prohibited from obtaining anti-coronavirus vaccinations. This is an advantageous feature that distinguishes this compound from other approved drugs. 

Rosmarinic acid, an ester of caffeic acid and 3,4-dihydroxy phenyl lactic acid, is present in most Lamiaceae species [46]. It has a broad inhibitory effect on a variety of viruses, e.g., rosmarinic acid inhibits HBV replication in HBV-infected cells by specifically targeting DNA polymerase ε binding [47]. It also inhibits influenza viruses and enterovirus 71 [48,49]. Although previous studies suggested that rosmarinic acid inhibits SARS-CoV-2 replication with an IC_50_ value of 25.47 ng/µL, the mechanism of action is still unknown [50]. Our results indicated that rosmarinic acid binds to SARS-CoV-2 M^pro^ with an IC_50_ value of 9.43 µM. Additionally, several animal studies revealed that rosmarinic acid has anti-inflammatory activity through the inhibition of NF-κB and STAT3 signaling pathways [51,52] and may be applied against arthritis, inflammatory bowel disease, and asthma [53]. Hence, rosmarinic acid is a potential therapeutic against COVID-19, especially for fighting immunological overreactions (i.e., the cytokine storm) during severe courses of the disease. 

Isorhamnetin is a flavonoid from *Hippophae rhamnoides* L. [54], *Artemisia absinthium* L. [55], and other plants. Isorhamnetin has a wide range of pharmacological effects on cardiovascular diseases, a variety of tumors, and neurodegenerative diseases [54]. Isorhamnetin exerts anti-influenza effects in vitro and in vivo by inhibition of hemagglutinin and neuraminidase [56]. Isorhamnetin also inhibits SARS-CoV-2 entry through inhibition of the Spike protein [57]. Our results showed that isorhamnetin binds and inhibits M^pro^ of SARS-CoV-2 and SARS-CoV-1 with IC_50_ values of 8.42 and 13.13 µM, respectively. Isorhamnetin has anti-inflammatory effects against different diseases, such as inflammatory bowel disease [58], osteoarthritis, and periodontitis, by suppressing the production of inflammatory mediators, cytokines, and reactive oxygen species [54]. Hence, targeting the Spike protein and M^pro^ makes isorhamnetin a promising drug candidate for the inhibition of coronavirus entry and replication. 

Luteolin is a natural flavonoid that is extensively present in many plant species [59]. It has multiple biological effects such as anti-inflammation, antiallergy, and anticancer activities [60]. In vivo and in vitro studies demonstrated that luteolin inhibits HBV replication through ERK-mediated downregulation of HNF4α expression [61]. It also exhibits antiviral activity against influenza A virus, HIV-1, and JEV [62,63,64]. Our results demonstrated that luteolin binds to SARS-CoV-2 M^pro^ and inhibits its activity with an IC_50_ value of 11.81 µM. Several in vivo and in vitro studies revealed that luteolin has an anti-inflammatory effect by blocking the NF-κB and AP-1 activation pathways [65,66,67,68]. Luteolin was also suggested as a potential therapeutic strategy for various autoimmune diseases, such as Hashimoto’s thyroiditis and multiple sclerosis [69,70]. Therefore, luteolin may be beneficial for COVID-19 patients with overshooting autoimmune reactions. 

In conclusion, we demonstrated that it is possible to identify natural products that exert activity against several coronaviruses and may be useful for developing pan-coronaviral drugs. There was some selectivity between the inhibition of coronaviral M^pro^ and cytotoxic activity toward human lung cells. Though the cytotoxicity was very low, the inhibitory rates toward the tested coronaviral main proteases were in the micromolar but not nanomolar range. Hence, animal experimentation should clarify whether this in vitro activity is reflected in vivo. Furthermore, the chemical scaffolds of the identified natural products may serve as lead structures when generating (semi)synthetic derivatives with improved activity. The concept of developing pan-coronaviral drugs is attractive for being prepared for future outbreaks of epidemic or pandemics by known or novel coronaviruses. 

## 4. Materials and Methods

### 4.1. Compounds

The chemical structures of natural products were downloaded from ZINC and PubChem databases in three-dimensional SDF format. Based on in silico studies, 12 selected compounds were provided by Fischer Analytics/Fischer Organics GmbH (Weiler, Germany). The compounds had a purity of >95%.

### 4.2. Virtual Screening

In this study, the PyRx software was used for the virtual screening of 39,442 natural-product-like compounds from the ZINC database and 121 natural compounds used in herbal medicines against viral diseases. As a target, the dimeric form of SARS-CoV-2 M^pro^ (PDB ID: 6XMk) was chosen to identify compounds with high binding affinity and low binding energy (kcal/mol). AutoDock version 1.5.6 was used to convert the Protein Data Bank files of target proteins (PDB) to PDBQT files. The energy of the compounds was minimized and converted from SDF format to PDBQT format using the PyRx software.

### 4.3. Sequence Alignment

The full-length amino acid sequences of M^pro^ of SARS-CoV-2, SARS-CoV-1, MERS-CoV, HCoV-HKU1, HCoV-NL63, HCoV-OC43, and HCoV-229E were accessed from the UniProt database and aligned by Clustal Omega; figures were prepared using Jalviwe 2.11.1.4.

### 4.4. Inhibition of M^pro^ Enzyme Activity

Enzymatic assays were performed using the SensoLyte SARS-CoV-2 3CL Protease Activity Assay Kit (AnaSpec, San Francisco, CA, USA), SARS-CoV-1 Assay Kit, and 3CL Protease MERS-CoV Assay Kit (BPS Bioscience, San Diego, CA, USA). Twelve selected compounds were diluted in assay buffer to a final concentration of 1 mM. Compound aliquots of 10 μL were added to black 96-well plates (Greiner, Frickenhausen, Germany), and 40 μL of 0.1 mg/mL M^pro^ was added to each well of the plates and incubated with the compounds at 37 °C for 30 min. The enzymatic reactions were initiated by adding a fluorescent substrate. The final concentration of the compound was 100 µM. Fluorescence was measured using an Infinite M2000 Pro plate reader (Tecan, Crailsheim, Germany). All values were subtracted from blank values. Then, compounds exhibiting more than 50% inhibitory activity at a fixed concentration of 100 µM were selected for dose-response studies in a concentration range from 0 to 100 µM for SARS-CoV-2, SARS-CoV-1, and MERS-CoV to calculate 50% inhibition concentrations (IC_50_). The activity percentage of M^pro^ was calculated using the following equation: Activity % = 100 − [(RFU_Vehicle control_ − RFU_tested sample_)/RFU_Vehicle control_ × 100].

### 4.5. Molecular Docking

The top compounds obtained from in vitro experiments were subjected to AutoDock 4.2.6 to identify their binding affinity to M^pro^ of SARS-CoV-2, SARS-CoV-1 (PDB ID: 6xhl), MERS-CoV (PDB ID: 6vh0), HCoV-HKU1 (PDB ID: 3d23), HCoV-NL63 (PDB ID: 3tlo), HCoV-OC43, and HCoV-229E (PDB ID: 2zu2). SWISS-MODEL was used to model the main protease structure. For this purpose, a Lamarckian algorithm was used with 250 runs and 2.5 million energy evaluations, as previously described in [71]. Docking was conducted using the high-performance supercomputer, MOGON II (Johannes Gutenberg University, Mainz, Germany). Three-dimensional illustrations of the compound–protein interactions were prepared using Molecular Dynamics (VMD) software.

### 4.6. Microscale Thermophoresis

We performed microscale thermophoresis (MST) to determine the dissociation constant (Kd) values for binding of hypericin, rosmarinic acid, isorhamnetin, and luteolin to recombinant M^pro^ proteins of SARS-CoV-2, SARS-CoV-1, and MERS-CoV (Bio-Techne, Wiesbaden, Germany). This method was performed as previously described in [71]. The three recombinant proteins were labeled with Monolith Protein Labeling Kit RED-NHS 2nd Generation (MO-L011, Nano Temper Technologies GmbH, Munich, Germany) according to the manufacturer’s instructions. The final protein concentrations after labeling were 3530, 882, and 910 nM for recombinant M^pro^ of SARS-CoV-2, SARS-CoV-1, and MERS-CoV, respectively. Titration was performed using a wide concentration range of compounds (dilution steps 1:1). The incubation time of ligand and protein was 30 min at room temperature in assay buffer (50 mM Tris buffer (pH 7.4) containing 10 mM MgCl_2_, 150 mM NaCl, and 0.05% Tween-20). Measurements were carried out in Monolith NT.115 standard capillaries (MO-K022, Nano Temper Technologies GmbH, Munich, Germany). Signals were measured using Monolith NT.115 instrument (Nano Temper Technologies) under the settings 40% LED power and 20, 10, and 40 MST power for recombinant M^pro^ proteins of SARS-CoV-2, SARS-CoV-1, and MERS-CoV, respectively. Fitting curves and Kd values were calculated by MO.Affinity Analysis software (Nano Temper Technologies).

### 4.7. Cell Viability Assay

Cell viability was measured using the resazurin assay as previously described in [72]. Human diploid MRC-5 lung fibroblasts was kindly provided by Dr. rer. nat. Sebastian Zahnreich (Department of Radiation Oncology and Radiation Therapy, University Medical Center of the Johannes Gutenberg University, Mainz, Germany) were seeded (5 × 10^5^ cells per well) into 96-well culture plates and incubated overnight before treatment. On the second day, the cells were treated with 10 concentrations of the four compounds in a range of 0.3–100 μM. After 72 h incubation, 20 μL 0.01% resazurin (Promega, Mannheim, Germany) was added to each well. Fluorescence was detected after 4 h incubation using an Infinite M2000 Pro plate reader (Tecan) at Ex/Em = 550 nm/590 nm wavelength. Cell viability was calculated in comparison to DMSO control. The DMSO final concentration was 0.5%. The 50% cytotoxicity concentration (CC_50_) values were calculated in comparison to the DMSO-treated control. Each experiment was independently repeated three times with six wells for each concentration. Therapeutic indices were calculated using the following equation: Therapeutic index = TD_50_/ED_50_.

## 5. Conclusions

**Figure 9:**
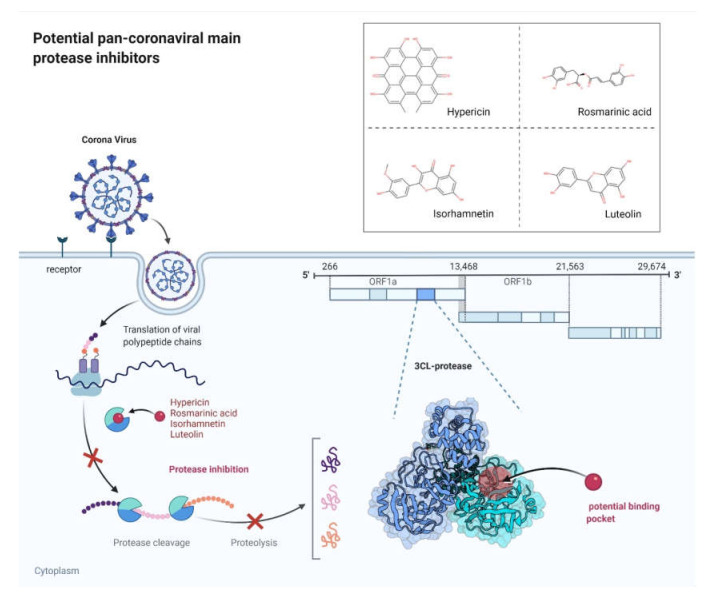
Potential pan-coronaviral M^pro^ inhibitors. Image adapted from BioRender.com (2022) with permission.

Overall, our in silico and in vitro results demonstrated that hypericin is a potential novel pan-anti-coronaviral agent as it binds to and inhibits M^pro^ of human-pathogenic coronaviruses. Moreover, isorhamnetin showed inhibitory effects toward SARS-CoV-2 and SARS-CoV-1 M^pro^, while luteolin revealed inhibitory effects against SARS-CoV-2 M^pro^ (Figure 9). Our results need to be further validated in animal models and clinical trials. A typical feature of natural products is that they are frequently multi-specific, i.e., they exert specific activities against several targets [73]. Therefore, the anti-coronaviral activity of the compounds we investigated is complemented with known anti-inflammatory effects reported in the literature. This may qualify these compounds not only to inhibit coronavirus replication but also to improve inflammatory conditions in severe courses of COVID-19 and other coronavirus infections. Natural products are generally considered to be of low toxicity. This is another property that speaks in favor of the four compounds we investigated.

## Figures and Tables

**Table 1:** Results of virtual screening with PyRx and molecular docking with AutoDock 4.2.6 of 12 selected compounds and GC376 as positive control binding to SARS-CoV-2 main protease.

Compound	PyRx Binding Affinity (kcal/mol)	Lowest Binding Energy (kcal/mol)	Predicted Ki (nM)
Hypericin	−8.70	−12.44 ± <0.01	0.762 ± 1.34
Curcumin	−7.90	−12.48 ± 0.04	0.679 ± 11.7
Isoliquiritin	−7.60	−11.69 ± 0.02	2.64 ± 0.11
Quercetin	−9.20	−10.72 ± 0.03	13.90 ± 0.80
Rosmarinic acid	−7.80	−9.98 ± 0.08	42.62 ± 3.25
Delphinidin	−8.50	−9.23 ± <0.01	170.92 ± 0.06
Cyanidin	−8.20	−9.13 ± <0.01	203.87 ± 0.04
Isorhamnetin	−8.20	−9.06 ± <0.01	237.99 ± 11.59
Luteolin	−8.10	−9.01 ± <0.01	247.73 ± 1.21
Kaempferol	−8.00	−8.77 ± <0.01	375.12 ± 0.05
Berberine	−8.10	−8.07 ± <0.01	1210 ± 0.01
Naringenin	−7.80	−8.00 ± 0.04	1370 ± 0.1
GC376 (positive control)	−8.00	−12.58 ± 0.29	0.70 ± 0.42

**Table 2:** Percent identity of SARS-CoV-2 M^pro^ with six human-pathogenic β-coronaviruses (complete protein sequence).

Human Coronavirus	% Identity with SARS-CoV-2
SARS-CoV-1	96.08
MERS-CoV	50.83
HcoV-NL63	49.17
HcoV-OC43	48.51
HcoV-HKU1	44.04
HcoV-229E	41.06

**Table 3:** IC_50_ values of selected compounds that inhibited M^pro^ of SARS-CoV-2, SARS-CoV-1, and MERS-CoV.

Compound		IC_50_ Value (µM) (mean ± SD)	
	SARS-CoV-2 M^pro^	SARS-CoV-1 M^pro^	MERS-CoV M^pro^
Hypericin	23.30 ± 1.21	19.43 ± 3.11	49.65 ± 5.41
Rosmarinic acid	9.43 ± 0.46	n.a.	n.a.
Isorhamnetin	8.42 ± 1.15	13.13 ± 1.78	n.a.
Luteolin	11.81 ± 1.27	n.a.	n.a.

**Table 4:** K_d_ values of selected compounds inhibiting main proteases of SARS-CoV-2, SARS-CoV-1, and MERS-CoV.

Compound		Kd Value (µM)	
	SARS-CoV-2 M^pro^	SARS-CoV-1 M^pro^	MERS-CoV M^pro^
Hypericin	7.73 ± 6.50	25.49 ± 13.61	54.91 ± 13.80
Rosmarinic acid	15.47 ± 4.77	n.a.	n.a.
Isorhamnetin	4.37 ± 3.90	3.60 ± 2.60	n.a.
Luteolin	13.41 ± 2.70	n.a.	n.a.

**Table 5:** Molecular docking of potential inhibitors and GC376 (positive control) to the catalytic center of main proteases of human-pathogenic coronaviruses.

Compound			Lowest Binding Energy (kcal/mol)				
	SARS-CoV-2	SARS-CoV-1	MERS-CoV	HCoV-HKU1	HCoV-NL63	HCoV-OC43	HCoV-229E
Hypericin	−12.44 ± <0.01	−11.53 ± 0.005	−11.98 ± 1.77	−9.11 ± < 0.01	−9.77 ± 0.31	−12.99 ± <0.01	−10.65 ± <0.01
Rosmarinic acid	−9.90 ± 0.08	−9.80 ± 0.03	−10.12 ± 0.21	−10.48 ± 0.31	−10.18 ± 0.11	−10.06 ± 0.06	−10.61 ± 0.05
Isorhamnetin	−9.06 ± <0.01	−8.83 ± <0.01	−8.59 ± <0.01	−8.50 ± <0.01	−8.57 ± <0.01	−8.33 ± <0.01	−8.19 ± 0.01
Luteolin	−9.01 ± <0.01	−7.66 ± <0.01	−7.67 ± 0.06	−7.65 ± <0.01	−9.25 ± <0.01	−8.21 ± <0.01	−8.02 ± <0.01
GC376 (positive control)	−12.58 ± 0.29	−12.17 ± 0.27	−13.65 ± 0.44	−12.78 ± 0.5	−11.04 ± 0.09	−12.28 ± 0.05	−11.52 ± 0.09

**Table 6:** Cytotoxicity of candidate compounds toward human fetal MRC-5 lung fibroblast cells as determined by the resazurin reduction assay.

Compound	CC_50_ Value (µM)
	(mean ± SD)
Hypericin	55.46 ± 2.2
Isorhamnetin	36.80 ± 3.4
Rosmarinic acid	n.a.
Luteolin	n.a.

**Table 7:** Therapeutic index values of hypericin and isorhamnetin.

Compound		Therapeutic Index	
	SARS-CoV-2 M^pro^	SARS-CoV-1 M^pro^	MERS-CoV M^pro^
Hypericin	2.38	2.85	1.11
Isorhamnetin	4.37	2.80	n.a.

## Data Availability

Data is contained within the article.

## References

[1] V’Kovski P., Kratzel A., Steiner S., Stalder H., Thiel V. (2021). Coronavirus biology and replication: Implications for SARS-CoV-2. Nat. Rev. Microbiol..

[2] Park S.E. (2020). Epidemiology, virology, and clinical features of severe acute respiratory syndrome -coronavirus-2 (SARS-CoV-2; Coronavirus Disease-19). Korean J. Pediatr..

[3] Totura A.L., Bavari S. (2019). Broad-spectrum coronavirus antiviral drug discovery. Expert Opin. Drug Discov..

[4] Xia S., Yan L., Xu W., Agrawal A.S., Algaissi A., Tseng C.-T.K., Wang Q., Du L., Tan W., Wilson I.A. (2019). A pan-coronavirus fusion inhibitor targeting the HR1 domain of human coronavirus spike. Sci. Adv..

[5] Menachery V.D., Yount B.L., Debbink K., Agnihothram S., Gralinski L.E., Plante J.A., Graham R.L., Scobey T., Ge X.-Y., Donaldson E.F. (2015). A SARS-like Cluster of Circulating Bat Coronaviruses Shows Potential for Human Emergence. Nat. Med..

[6] Xiao K., Zhai J., Feng Y., Zhou N., Zhang X., Zou J.-J., Li N., Guo Y., Li X., Shen X. (2020). Isolation of SARS-CoV-2-related coronavirus from Malayan pangolins. Nature.

[7] Wacharapluesadee S., Tan C.W., Maneeorn P., Duengkae P., Zhu F., Joyjinda Y., Kaewpom T., Ni Chia W., Ampoot W., Lim B.L. (2021). Evidence for SARS-CoV-2 related coronaviruses circulating in bats and pangolins in Southeast Asia. Nat. Commun..

[8] Grange Z.L., Goldstein T., Johnson C.K., Anthony S., Gilardi K., Daszak P., Olival K.J., O’Rourke T., Murray S., Olson S.H. (2021). Ranking the risk of animal-to-human spillover for newly discovered viruses. Proc. Natl. Acad. Sci. USA.

[9] Johnson C.K., Hitchens P.L., Pandit P.S., Rushmore J., Evans T.S., Young C.C.W., Doyle M.M. (2020). Global shifts in mammalian population trends reveal key predictors of virus spillover risk. Proc. R. Soc. B Biol. Sci..

[10] Lu R., Zhao X., Li J., Niu P., Yang B., Wu H., Wang W., Song H., Huang B., Zhu N. (2020). Genomic characterisation and epidemiology of 2019 novel coronavirus: Implications for virus origins and receptor binding. Lancet.

[11] Harrison A.G., Lin T., Wang P. (2020). Mechanisms of SARS-CoV-2 Transmission and Pathogenesis. Trends Immunol..

[12] Hu B., Guo H., Zhou P., Shi Z.-L. (2021). Characteristics of SARS-CoV-2 and COVID-19. Nat. Rev. Microbiol..

[13] Kneller D.W., Phillips G., O’Neill H.M., Jedrzejczak R., Stols L., Langan P., Joachimiak A., Coates L., Kovalevsky A. (2020). Structural plasticity of SARS-CoV-2 3CL Mpro active site cavity revealed by room temperature X-ray crystallography. Nat. Commun..

[14] Jin Z., Du X., Xu Y., Deng Y., Liu M., Zhao Y., Zhang B., Li X., Zhang L., Peng C. (2020). Structure of Mpro from SARS-CoV-2 and discovery of its inhibitors. Nature.

[15] Ma C., Sacco M.D., Hurst B., Townsend J.A., Hu Y., Szeto T., Zhang X., Tarbet B., Marty M., Chen Y. (2020). Boceprevir, GC-376, and calpain inhibitors II, XII inhibit SARS-CoV-2 viral replication by targeting the viral main protease. Cell Res..

[16] Ullrich S., Nitsche C. (2020). The SARS-CoV-2 main protease as drug target. Bioorg. Med. Chem. Lett..

[17] Hilgenfeld R. (2014). From SARS to MERS: Crystallographic studies on coronaviral proteases enable antiviral drug design. FEBS J..

[18] Lipinski C.A., Lombardo F., Dominy B.W., Feeney P.J. (2001). Drug Delivery Reviews Experimental and computational approaches to estimate solubility and permeability in drug discovery and development settings. Adv. Drug Deliv. Rev..

[19] Musarra-Pizzo M., Pennisi R., Ben-Amor I., Mandalari G., Sciortino M. (2021). Antiviral Activity Exerted by Natural Products against Human Viruses. Viruses.

[20] Koehn F.E., Carter G.T. (2005). The evolving role of natural products in drug discovery. Nat. Rev. Drug Discov..

[21] Kadioglu O., Saeed M., Greten H.J., Efferth T. (2021). Identification of novel compounds against three targets of SARS CoV-2 coronavirus by combined virtual screening and supervised machine learning. Comput. Biol. Med..

[22] Miskovsky P. (2002). Hypericin—A New Antiviral and Antitumor Photosensitizer: Mechanism of Action and Interaction with Biological Macromolecules. Curr. Drug Targets.

[23] Fukuchi K., Okudaira N., Adachi K., Odai-Ide R., Watanabe S., Ohno H., Yamamoto M., Kanamoto T., Terakubo S., Nakashima H. (2016). Antiviral and Antitumor Activity of Licorice Root Extracts. In Vivo.

[24] Jennings M., Parks R. (2020). Curcumin as an Antiviral Agent. Viruses.

[25] Vázquez-Calvo A., de Oya N.J., Martin-Acebes M.A., Garcia-Moruno E., Saiz J.-C. (2017). Antiviral Properties of the Natural Polyphenols Delphinidin and Epigallocatechin Gallate against the Flaviviruses West Nile Virus, Zika Virus, and Dengue Virus. Front. Microbiol..

[26] Cataneo A.H.D., Kuczera D., Koishi A.C., Zanluca C., Silveira G.F., De Arruda T.B., Suzukawa A.A., Bortot L.O., Dias-Baruffi M., Verri W.A. (2019). The citrus flavonoid naringenin impairs the in vitro infection of human cells by Zika virus. Sci. Rep..

[27] Kai H., Obuchi M., Yoshida H., Watanabe W., Tsutsumi S., Park Y.K., Matsuno K., Yasukawa K., Kurokawa M. (2014). In vitro and in vivo anti-influenza virus activities of flavonoids and related compounds as components of Brazilian propolis (AF-08). J. Funct. Foods.

[28] Cheng S.-C., Chang G.-G., Chou C.-Y. (2010). Mutation of Glu-166 Blocks the Substrate-Induced Dimerization of SARS Coronavirus Main Protease. Biophys. J..

[29] Zhang L., Lin D., Sun X., Curth U., Drosten C., Sauerhering L., Becker S., Rox K., Hilgenfeld R. (2020). Crystal structure of SARS-CoV-2 main protease provides a basis for design of improved α-ketoamide inhibitors. Science.

[30] Tomar S., Johnston M.L., John S.E.S., Osswald H.L., Nyalapatla P.R., Paul L.N., Ghosh A.K., Denison M.R., Mesecar A.D. (2015). Ligand-induced Dimerization of Middle East Respiratory Syndrome (MERS) Coronavirus nsp5 Protease (3CLpro): Implications for Nsp5 Regulation and the Development of Antivirals. J. Biol. Chem..

[31] Ho B.-L., Cheng S.-C., Shi L., Wang T.-Y., Ho K.-I., Chou C.-Y. (2015). Critical Assessment of the Important Residues Involved in the Dimerization and Catalysis of MERS Coronavirus Main Protease. PLoS ONE.

[32] Khan S.A., Zia K., Ashraf S., Uddin R., Ul-Haq Z. (2021). Identification of chymotrypsin-like protease inhibitors of SARS-CoV-2 via integrated computational approach. J. Biomol. Struct. Dyn..

[33] Lu I.-L., Mahindroo N., Liang P.-H., Peng Y.-H., Kuo C.-J., Tsai K.-C., Hsieh H.-P., Chao Y.-S., Wu S.-Y. (2006). Structure-Based Drug Design and Structural Biology Study of Novel Nonpeptide Inhibitors of Severe Acute Respiratory Syndrome Coronavirus Main Protease. J. Med. Chem..

[34] Birt D.F., Widrlechner M.P., Hammer K.D.P., Hillwig M.L., Wei J., Kraus G.A., Murphy P.A., McCoy J.A., Wurtele E.S., Neighbors J.D. (2009). Hypericum in Infection: Identification of Anti-Viral and Anti-Inflammatory Constituents. Pharm. Biol..

[35] Chen H., Feng R., Muhammad I., Abbas G., Zhang Y., Ren Y., Huang X., Zhang R., Diao L., Wang X. (2019). Protective effects of hypericin against infectious bronchitis virus induced apoptosis and reactive oxygen species in chicken embryo kidney cells. Poult. Sci..

[36] Shih C.-M., Wu C.-H., Wu W.-J., Hsiao Y.-M., Ko J.-L. (2018). Hypericin inhibits hepatitis C virus replication via deacetylation and down-regulation of heme oxygenase-1. Phytomedicine.

[37] Jacobson J.M., Feinman L., Liebes L., Ostrow N., Koslowski V., Tobia A., Cabana B.E., Lee D.-H., Spritzler J., Prince A.M. (2001). Pharmacokinetics, Safety, and Antiviral Effects of Hypericin, a Derivative of St. John’s Wort Plant, in Patients with Chronic Hepatitis C Virus Infection. Antimicrob. Agents Chemother..

[38] Lavie G., Valentine F., Levin B., Mazur Y., Gallo G., Lavie D., Weiner D., Meruelo D. (1989). Studies of the mechanisms of action of the antiretroviral agents hypericin and pseudohypericin. Proc. Natl. Acad. Sci. USA.

[39] Xu Y. (2005). Raman spectroscopic study on structure of human immu-nodeficiency virus (HIV) and hypericin-induced photosen-sitive damage of HIV. Sci. China Ser. C Life Sci..

[40] Gulick R.M., McAuliffe V., Holden-Wiltse J., Crumpacker C., Liebes L., Stein D.S., Meehan P., Hussey S., Forcht J., Valentine F.T. (1999). Phase I Studies of Hypericin, the Active Compound in St. John’s Wort, as an Antiretroviral Agent in HIV-Infected Adults: AIDS Clinical Trials Group Protocols 150 and 258. Ann. Intern. Med..

[41] Zhang Y., Chen H., Zou M., Oerlemans R., Shao C., Ren Y., Zhang R., Huang X., Li G., Cong Y. (2021). Hypericin Inhibit Alpha-Coronavirus Replication by Targeting 3CL Protease. Viruses.

[42] Pitsillou E., Liang J., Karagiannis C., Ververis K., Darmawan K.K., Ng K., Hung A., Karagiannis T.C. (2020). Interaction of small molecules with the SARS-CoV-2 main protease in silico and in vitro validation of potential lead compounds using an enzyme-linked immunosorbent assay. Comput. Biol. Chem..

[43] Dellafiora L., Galaverna G., Cruciani G., Dall’Asta C., Bruni R. (2018). On the Mechanism of Action of Anti-Inflammatory Activity of Hypericin: An In Silico Study Pointing to the Relevance of Janus Kinases Inhibition. Molecules.

[44] Zhang K., Gao S., Guo J., Ni G., Chen Z., Li F., Zhu X., Wen Y., Guo Y. (2018). Hypericin-photodynamic therapy inhibits proliferation and induces apoptosis in human rheumatoid arthritis fibroblast-like synoviocytes cell line MH7A. Iran. J. Basic Med. Sci..

[45] Perinbam K., Mahendrakumar M., Seeni S. (2018). Hypericin, an anthraquinone derivative of Hypericum hookerianum wight and Arn. (Hypericaceae) of Palni Hills, South India, exhibits anti-inflammatory property in lipopolysaccharide—stimulated raw 264.7 macrophages. Pharmacogn. Mag..

[46] Levsh O., Pluskal T., Carballo V., Mitchell A.J., Weng J.-K. (2019). Independent evolution of rosmarinic acid biosynthesis in two sister families under the Lamiids clade of flowering plants. J. Biol. Chem..

[47] Tsukamoto Y., Ikeda S., Uwai K., Taguchi R., Chayama K., Sakaguchi T., Narita R., Yao W.-L., Takeuchi F., Otakaki Y. (2018). Rosmarinic acid is a novel inhibitor for Hepatitis B virus replication targeting viral epsilon RNA-polymerase interaction. PLoS ONE.

[48] Mahalapbutr P., Sangkhawasi M., Kammarabutr J., Chamni S., Rungrotmongkol T. (2020). Rosmarinic Acid as a Potent Influenza Neuraminidase Inhibitor: In Vitro and In Silico Study. Curr. Top. Med. Chem..

[49] Hsieh C.-F., Jheng J.-R., Lin G.-H., Chen Y.-L., Ho J.-Y., Liu C.-J., Hsu K.-Y., Chen Y.-S., Chan Y.F., Yu H.-M. (2020). Rosmarinic acid exhibits broad anti-enterovirus A71 activity by inhibiting the interaction between the five-fold axis of capsid VP1 and cognate sulfated receptors. Emerg. Microbes Infect..

[50] Elebeedy D., Elkhatib W.F., Kandeil A., Ghanem A., Kutkat O., Alnajjar R., Saleh M.A., El Maksoud A.I.A., Badawy I., Al-Karmalawy A.A. (2021). Anti-SARS-CoV-2 activities of tanshinone IIA, carnosic acid, rosmarinic acid, salvianolic acid, baicalein, and glycyrrhetinic acid between computational and in vitro insights. RSC Adv..

[51] Jiang K., Ma X., Guo S., Zhang T., Zhao G., Wu H., Wang X., Deng G. (2017). Anti-inflammatory Effects of Rosmarinic Acid in Lipopolysaccharide-Induced Mastitis in Mice. Inflammation.

[52] Jin B.-R., Chung K.-S., Cheon S.-Y., Lee M., Hwang S., Hwang S.N., Rhee K.-J., An H.-J. (2017). Rosmarinic acid suppresses colonic inflammation in dextran sulphate sodium (DSS)-induced mice via dual inhibition of NF-κB and STAT3 activation. Sci. Rep..

[53] Luo C., Zou L., Sun H., Peng J., Gao C., Bao L., Ji R., Jin Y., Sun S. (2020). A Review of the Anti-Inflammatory Effects of Rosmarinic Acid on Inflammatory Diseases. Front. Pharmacol..

[54] Gong G., Guan Y.-Y., Zhang Z.-L., Rahman K., Wang S.-J., Zhou S., Luan X., Zhang H. (2020). Isorhamnetin: A review of pharmacological effects. Biomed. Pharmacother..

[55] Haghi G., Safaei A., Ghomi J.S. (2004). Identification and determination of flavonoids in leaf, dried aqueous and dried hydroalcoholic extract of Artemisia absinthium by HPLC. Iran. J. Basic Med. Sci..

[56] Dayem A.A., Choi H.Y., Kim Y.B., Cho S.-G. (2015). Antiviral Effect of Methylated Flavonol Isorhamnetin against Influenza. PLoS ONE.

[57] Zhan Y., Ta W., Tang W., Hua R., Wang J., Wang C., Lu W. (2021). Potential antiviral activity of isorhamnetin against SARS-CoV-2 spike pseudotyped virus in vitro. Drug Dev. Res..

[58] Dou W., Zhang J., Li H., Kortagere S., Sun K., Ding L., Ren G., Wang Z., Mani S. (2014). Plant flavonol isorhamnetin attenuates chemically induced inflammatory bowel disease via a PXR-dependent pathway. J. Nutr. Biochem..

[59] Imran M., Rauf A., Abu-Izneid T., Nadeem M., Shariati M.A., Khan I.A., Imran A., Orhan I.E., Rizwan M., Atif M. (2019). Luteolin, a flavonoid, as an anticancer agent: A review. Biomed. Pharmacother..

[60] Lin Y., Shi R., Wang X., Shen H.-M. (2008). Luteolin, a Flavonoid with Potential for Cancer Prevention and Therapy. Curr. Cancer Drug Targets.

[61] Bai L., Nong Y., Shi Y., Liu M., Yan L., Shang J., Huang F., Lin Y., Tang H. (2015). Luteolin Inhibits Hepatitis B Virus Replication through Extracellular Signal-Regulated Kinase-Mediated Down-Regulation of Hepatocyte Nuclear Factor 4α Expression. Mol. Pharm..

[62] Fan W., Qian S., Qian P., Li X. (2016). Antiviral activity of luteolin against Japanese encephalitis virus. Virus Res..

[63] Yan H., Ma L., Wang H., Wu S., Huang H., Gu Z., Jiang J., Li Y. (2019). Luteolin decreases the yield of influenza A virus in vitro by interfering with the coat protein I complex expression. J. Nat. Med..

[64] Mehla R., Bivalkar-Mehla S., Chauhan A. (2011). A Flavonoid, Luteolin, Cripples HIV-1 by Abrogation of Tat Function. PLoS ONE.

[65] Ueda H., Yamazaki C., Yamazaki M. (2002). Luteolin as an Anti-Inflammatory and Anti-Allergic Constituent of Perilla. Biol. Pharm. Bull..

[66] Chen C.-Y., Peng W.-H., Tsai K.-D., Hsu S.-L. (2007). Luteolin suppresses inflammation-associated gene expression by blocking NF-κB and AP-1 activation pathway in mouse alveolar macrophages. Life Sci..

[67] Franza L., Carusi V., Nucera E., Pandolfi F. (2021). Luteolin, inflammation and cancer: Special emphasis on gut microbiota. BioFactors.

[68] Theoharides T.C. (2009). Luteolin as a therapeutic option for multiple sclerosis. J. Neuroinflamm..

[69] Xia N., Chen G., Liu M., Ye X., Pan Y., Ge J., Mao Y., Wang H., Wang J., Xie S. (2016). Anti-inflammatory effects of luteolin on experimental autoimmune thyroiditis in mice. Exp. Ther. Med..

[70] Zeino M., Saeed M.E.M., Kadioglu O., Efferth T. (2014). The ability of molecular docking to unravel the controversy and challenges related to P-glycoprotein—a well-known, yet poorly understood drug transporter. Investig. New Drugs.

[71] Abdelfatah S., Berg A., Böckers M., Efferth T. (2019). A selective inhibitor of the Polo-box domain of Polo-like kinase 1 identified by virtual screening. J. Adv. Res..

[72] Abdelfatah S., Fleischer E., Klinger A., Wong V.K.W., Efferth T. (2019). Identification of inhibitors of the polo-box domain of polo-like kinase 1 from natural and semisynthetic compounds. Investig. New Drugs.

[73] Efferth T. (2011). Complex Interactions between Phytochemicals. The Multi-Target Therapeutic Concept of Phytotherapy. Curr. Drug Targets.

